# Choice of Empirical Antibiotic Therapy and Adverse Outcomes in Older Adults With Suspected Urinary Tract Infection: Cohort Study

**DOI:** 10.1093/ofid/ofz039

**Published:** 2019-01-18

**Authors:** Haroon Ahmed, Daniel Farewell, Nick A Francis, Shantini Paranjothy, Christopher C Butler

**Affiliations:** 1 Division of Population Medicine, Cardiff University School of Medicine, Neuadd Meirionnydd, Heath Park, United Kingdom; 2 Nuffield Department of Primary Care Health Sciences, University of Oxford, Radcliffe Primary Care Building, Radcliffe Observatory Quarter, United Kingdom

**Keywords:** aged, electronic health records, primary care, urinary tract infection

## Abstract

**Background:**

Nitrofurantoin is widely recommended for empirical treatment of urinary tract infection (UTI), but primary care clinicians may prescribe alternative antibiotics to improve prognosis in older, sicker patients. We assessed whether prescribing alternative antibiotics was associated with reduced risk of adverse outcomes in older patients.

**Methods:**

This retrospective cohort study included patients aged ≥65 years empirically treated for a UTI with nitrofurantoin, cefalexin, ciprofloxacin, or co-amoxiclav. We matched patients on their propensity to receive a nitrofurantoin prescription and used mixed-effects logistic regression to estimate odds ratios (ORs) and 95% confidence intervals (CIs) for reconsultation and represcription (proxy for treatment failure), hospitalization for UTI, sepsis, or acute kidney injury, and death.

**Results:**

We identified 42 298 patients aged ≥65 years prescribed empirical nitrofurantoin, cefalexin, ciprofloxacin, or co-amoxiclav for a UTI. Compared with nitrofurantoin, patients prescribed cefalexin, ciprofloxacin, or co-amoxiclav had lower odds of reconsultation and represcription (OR for cefalexin = 0.85, 95% CI = 0.75–0.98; OR for ciprofloxacin = 0.48, 95% CI = 0.38–0.61, OR for co-amoxiclav = 0.77, 95% CI = 0.64–0.93). Patients prescribed cefalexin or ciprofloxacin had greater odds of hospitalization for sepsis (OR for cefalexin = 1.89, 95% CI = 1.03–3.47; OR for ciprofloxacin = 3.21, 95% CI = 1.59–6.50), and patients prescribed cefalexin had greater odds of death (OR = 1.44, 95% CI = 1.12–1.85).

**Conclusions:**

Compared with nitrofurantoin, prescribing of alternative antibiotics for UTI in older people may be associated with lower rates of treatment failure but was not associated with reduced risk of UTI-related hospitalization or death.

Urinary tract infection (UTI) is the most common indication for antibiotic prescribing in older adults presenting to ambulatory care services [1] and those in long-term care facilities [2–4]. Approximately 60%–75% of adults presenting with suspected UTI receive empirical antibiotic therapy at the same consultation, without knowledge of microbiological susceptibilities [5–7]. Current clinical guidelines in the United States [8] and United Kingdom [9] recommend nitrofurantoin for empirical treatment of uncomplicated UTI. However, previous research found that approximately 15% of older adults empirically treated for a UTI in primary care were prescribed cefalexin, ciprofloxacin, or co-amoxiclav [10]. Cefalexin, ciprofloxacin, and co-amoxiclav are broad-spectrum antibiotics, and they are associated with increased rates of drug-related adverse events [11] and antibiotic-associated diarrhoea [12]. They are also more likely to select for drug-resistant organisms leading to subsequent antibiotic-resistant colonization or infection [8]. Qualitative research found that primary care clinicians were more likely to consider broad-spectrum antibiotics for older patients, who were frail, had comorbidities, and were judged to have more severe illness [13]. The perceived aim of broad-spectrum antibiotic prescribing was to prevent treatment failure, worsening illness, and hospitalization, events thought to be more likely if narrow-spectrum antibiotics were prescribed for that clinical scenario [13].

Meta-analysis of 3 randomized trials (n = 289) found similar clinical cure rates between patients with UTI treated with nitrofurantoin versus flouroquinolones, suggesting that flouroquinolones offer little additional benefit [14]. However, trials only included young, healthy women and were underpowered to assess risk of important but rare outcomes such as UTI-related hospitalization or death [15–17]. Previous observational studies have compared trimethoprim-sulfamethoxazole with flouroquinolones, and sulfamethiazole with pivmecillinam, but no trials or observational studies have compared nitrofurantoin with cefalexin or co-amoxiclav [18, 19].

Therefore, we used data from anonymized linked health records to compare the risk of adverse outcomes in adults aged ≥65 prescribed empirical nitrofurantoin versus cefalexin, ciprofloxacin, or co-amoxiclav for suspected UTI in primary care. Our aim was to assess whether cefalexin, ciprofloxacin, or co-amoxiclav were associated with a reduced risk of treatment failure, hospitalization for UTI, sepsis or acute kidney injury (AKI), or death. If these antibiotics were associated with risks that were similar or higher than those of nitrofurantoin, then this would support further reductions in their use, even in older, frailer, comorbid patients with more severe presenting features.

## METHODS

### Data Source

We used the Clinical Practice Research Datalink (CPRD), an electronic database of anonymized primary care records, covering 11.3 million patients from 674 general practices across the United Kingdom (UK) [20]. Approximately 7% of the UK population are included, and patients are broadly representative of the wider UK population in terms of age, gender, and ethnicity. The CPRD holds data on demographics, clinical encounters and diagnoses (coded using Read codes), drug prescriptions, blood tests, and referrals to specialists. Data are available once they have met a series of quality checks on completeness and reliability, and the CPRD deems them to be of the standard required for research purposes. Linked hospital and death registration data are available for patients from approximately 50% of contributing English practices. Hospital diagnoses and causes of death are recorded using version 10 of the *International Classification of Disease* (ICD-10).

The CPRD Independent Scientific Advisory Committee approved the study protocol (protocol number 17_250). Further ethical approval was not required because the proposed research was within the remit of the CPRD’s broad National Research Ethics Service approval. We used the Reporting of Studies Conducted using Observational Routinely-collected Health Data (RECORD) statement and checklist to guide study reporting [21].

### Design and Participants

This was a retrospective cohort study using linked health record data. Patients were eligible for inclusion if, between January 1, 2010 and December 31, 2016, their data were of the quality required by CPRD, they were ≥65 years old, and eligible for data linkage. Only patients registered with practices that consented to data linkage had linked hospital and death registry data. We excluded patients if they were temporary residents or had gaps in their data coverage. Follow-up began from the latest of, study start date (January 1, 2010), patient’s 65th birthday, 6 months after they registered with the practice (to avoid including historical UTIs recorded at registration), or the date their practice met the CPRD data quality requirements. Follow-up ended on the earliest of study end date (December 31, 2016), the day the patient died or transferred out of the practice (ie, last date of CPRD data collection), or 28 days after an incident UTI event. We identified eligible patients with (1) a Read code indicating an incident primary care presentation with a suspected UTI (codes available in Supplementary Appendix 1) and (2) a same-day prescription code indicating empirical prescribing of a relevant antibiotic. We defined “incident” as a consultation occurring in a patient without a UTI-related Read code or trimethoprim or nitrofurantoin prescription in the preceding 90 days (trimethoprim and nitrofurantoin are used almost exclusively for UTI in the UK). We used the first incident episode during each patient’s follow-up period. We excluded UTI episodes with a hospital discharge in the preceding 14 days to exclude hospital-acquired infections.

### Exposures

The exposure variable was the recorded empirical antibiotic prescription.

### Outcomes

We estimated risk of the following adverse outcomes for patients empirically treated in primary care for an incident suspected UTI: (1) reconsultation for urinary symptoms and a same-day antibiotic represcription within 14 days after the incident UTI, as a proxy for treatment failure, ascertained through Read and prescription codes recorded in primary care records; (2) hospitalization for UTI, sepsis, or AKI within 14 days after the incident UTI ascertained from ICD-10 codes recorded in linked hospital admission data for the first episode of a hospital admission, ie, the episode most likely responsible for the admission; (3) death within 28 days after the incident UTI using linked death registration data.

We chose 14 days for the reconsultation and hospitalization outcomes to increase the likelihood that these events were related to the initial UTI. Longer time periods increase the likelihood that the outcome may have been influenced by an intervening event, eg, if a 28-day period was used, a patient could have a UTI, recover, have a cardiac event, and be hospitalized with AKI. We chose 28 days for the death outcome because the UTI could precipitate events (eg, sepsis) that take some time to evolve before death.

### Statistical Analyses

We used primary care demographic and clinical codes to describe baseline characteristics for patients by prescribed antibiotic. To compare outcomes between patients prescribed nitrofurantoin versus cefalexin, ciprofloxacin, or co-amoxiclav, we matched patients on their propensity to receive a nitrofurantoin prescription. Variables included in the logistic regression models that generated the propensity score were age, Index of Multiple Deprivation score quintile [22], Charlson score [23], the presence or absence of a Read code indicating coronary heart disease, renal disease, respiratory disease, type 2 diabetes mellitus, heart failure, peripheral arterial disease, and stroke, because these variables were previously shown to be associated with antibiotic prescribing [7, 24]. We also included gender, whether the patient was housebound, had dementia, liver disease, rheumatoid arthritis, cancer, urinary incontinence or a urinary catheter, an estimated glomerular filtration rate, and polypharmacy (defined as records indicating ≥5 long-term medications per month in the year before the incident UTI), because these variables could be associated with both the clinical decision around choice of antibiotic and the UTI-related outcomes.

We used nearest neighbor matching with no replacement to match 3 patients with nitrofurantoin prescriptions to 1 patient with a cefalexin prescription. We assessed balance in measured baseline covariates between matched groups (1) by visually inspecting jitter plots and histograms of covariate distribution before and after matching and (2) by calculating standardized mean differences for covariates between groups. We regarded standardized mean differences of <0.1 as reflecting adequate balance [25, 26]. We used mixed-effects logistic regression [27] to calculated odds ratios (ORs) and 95% confidence intervals (CIs) for each outcome, accounting for clustering by general practice. We repeated the analyses by matching 3 patients with nitrofurantoin prescriptions to 1 patient with a ciprofloxacin prescription and then 3 patients with nitrofurantoin prescriptions to 1 patient with a co-amoxiclav prescription.

## RESULTS

**Figure 1.**
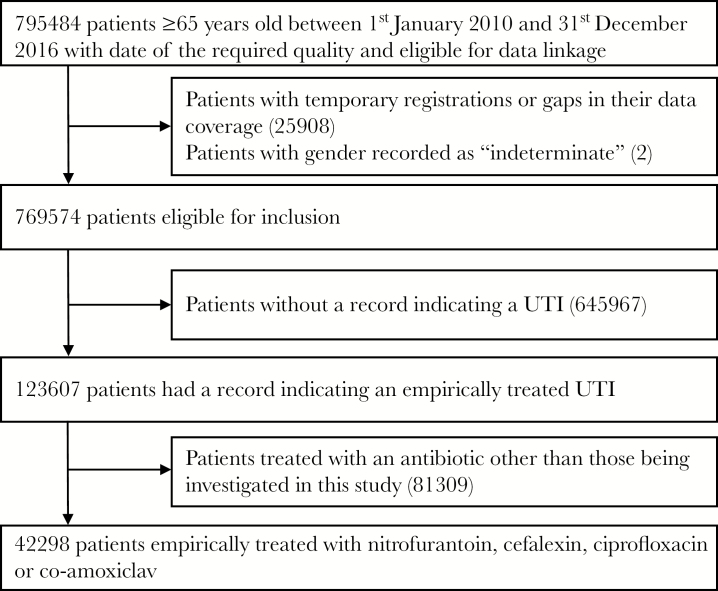
Flow of patients from initial identification in the database through to final cohort.

From a cohort of 795 484 patients aged 65 and over, we identified 123 607 (16%) with an incident empirically treated UTI, 42 298 of whom were prescribed nitrofurantoin, cefalexin, ciprofloxacin, or co-amoxiclav (Figure 1). In this final cohort, 11 420 (27%) patients were male, and the median age at time of incident UTI was 76 years (interquartile range, 70–83). Nitrofurantoin was the most commonly prescribed antibiotic, accounting for 60% of all prescriptions, followed by cefalexin (18%), co-amoxiclav (13%), and ciprofloxacin (9%).

### Baseline Characteristics

There were differences in baseline characteristics across the antibiotic groups. For example, 55% of the ciprofloxacin group were male compared with 23% of the nitrofurantoin group (Table 1). Compared with the nitrofurantoin group, greater proportions of the cefalexin, ciprofloxacin, and co-amoxiclav groups had comorbidities, particularly ischemic heart disease, heart failure, and renal disease. Approximately 3% of the nitrofurantoin group had a Charlson score of ≥6, compared with 5%–6% of the other groups.

**Table 1.** Baseline Characteristics by Prescribed Antibiotic^a^

Characteristic	Cefalexin	Ciprofloxacin	Co-amoxiclav	Nitrofurantoin
N	7546 (17.8)	3868 (9.1)	5516 (13.0)	25 368 (60.0)
Men	2150 (28.5)	2115 (54.7)	2229 (40.4)	5930 (23.4)
Mean (SD) age	77.5 (8.4)	76.5 (8.3)	77.6 (8.5)	76.5 (8.4)
Mean (SD) follow-up time (years)	4.2 (2.0)	4.5 (2.0)	4.2 (2.0)	4.6 (1.9)
Mean (SD) prescription duration (days)	8.2 (7.0)	7.6 (7.0)	8.3 (8.6)	6.6 (3.6)
Index of Multiple Deprivation Decile				
1 or 2 (least deprived)	1708 (22.6)	1056 (27.3)	1354 (24.5)	6850 (27.0)
3 or 4	1710 (22.7)	957 (24.7)	1401 (25.4)	6180 (24.4)
5 or 6	1722 (22.8)	850 (22.0)	1255 (22.8)	5214 (20.6)
7 or 8	1254 (16.6)	605 (15.6)	849 (15.4)	3970 (15.6)
9 or 10 (most deprived)	1152 (15.3)	400 (10.3)	657 (11.9)	3154 (12.4)
Housebound	41 (5.5)	136 (3.5)	265 (4.8)	929 (3.7)
Respiratory disease	1702 (22.6)	849 (21.9)	1198 (21.7)	5339 (21.0)
Cardiac failure	516 (6.8)	212 (5.5)	347 (6.3)	1083 (4.3)
Dementia	512 (6.8)	170 (4.4)	382 (6.9)	1439 (5.7)
Peripheral vascular disease	488 (6.5)	252 (6.5)	321 (5.8)	1082 (4.3)
Renal disease	2243 (29.7)	1001 (25.9)	1499 (27.2)	5310 (20.9)
Rhuematoid arthritis	297 (3.9)	108 (2.8)	188 (3.4)	900 (3.5)
Cancer	1295 (17.2)	780 (20.2)	949 (17.2)	3889 (15.3)
Stroke	886 (11.7)	392 (10.1)	673 (12.2)	2460 (9.7)
Diabetes	1474 (19.5)	783 (20.2)	1111 (20.1)	4234 (16.7)
Liver disease	68 (0.9)	30 (0.8)	36 (0.7)	171 (0.7)
Ischaemic heart disease	1602 (21.2)	821 (21.2)	1158 (21.0)	4290 (16.9)
Urinary catheter	372 (4.9)	309 (8.0)	327 (5.9)	853 (3.4)
Urinary incontinence	1199 (15.9)	471 (12.2)	867 (15.7)	3972 (15.7)
Polypharmacy	3299 (43.7)	1540 (39.8)	2376 (43.1)	9301 (36.7)
eGFR				
60–90	4168 (55.2)	2344 (60.6)	3170 (57.5)	16 719 (65.9)
45–59	1749 (23.2)	811 (21.0)	1227 (22.2)	5237 (20.6)
30–44	917 (12.2)	388 (10.0)	613 (11.1)	1815 (7.2)
15–29	319 (4.2)	148 (3.8)	208 (3.8)	391 (1.5)
<15	47 (0.6)	26 (0.7)	44 (0.8)	41 (0.2)
Missing	346 (4.6)	151 (3.9)	254 (4.6)	1165 (4.6)
Charlson Score				
0	2029 (26.9)	1073 (27.7)	1591 (28.8)	8845 (34.9)
1	1515 (20.1)	765 (19.8)	1098 (1909)	8845 (34.9)
2	1406 (18.6)	773 (20.0)	1006 (18.2)	4755 (18.7)
3	1070 (14.2)	535 (13.8)	769 (13.9)	3050 (12.0)
4	658 (8.7)	313 (8.1)	437 (7.9)	1567 (6.2)
5	428 (5.7)	197 (5.1)	305 (5.5)	955 (3.8)
≥6	440 (5.8)	212 (5.5)	310 (5.6)	842 (3.3)

Abbreviations: eGFR, estimated glomerular filtration rate; SD, standard deviation.

^a^Values are numbers (%) unless otherwise stated.

### Propensity-Score Matching

We matched 21 600 patients prescribed nitrofurantoin with 7200 patients prescribed cefalexin, 11 151 patients prescribed nitrofurantoin with 3717 patients prescribed ciprofloxacin, and 15 786 patients prescribed nitrofurantoin with 5262 patients prescribed co-amoxiclav (Table 2). Inspection of jitter plots and histograms suggested matching had improved balance of covariates across the 2 groups. Standardized mean differences were all less than 0.1.

**Table 2.** Propensity-Score Matched Analyses Comparing Outcomes Between Nitrofurantoin and Other Antibiotics

Outcomes	Cefalexin (n = 7200) Versus Nitrofurantoin (n = 21 600)		Ciprofloxacin (n = 3717) Versus Nitrofurantoin (n = 11 151)		Co-amoxiclav (n = 5262) Versus Nitrofurantoin (n = 15 786)	
	OR (95% CI)	*P* Value	OR (95% CI)	*P* Value	OR (95% CI)	*P* Value
Reconsultation and represcription within 14 days	0.85 (0.75–0.98)	.020	0.48 (0.38–0.61)	<.001	0.77 (0.64–0.93)	.006
Hospitalized for UTI within 14 days	0.96 (0.78–1.18)	.673	0.84 (0.57–1.26)	.408	0.94 (0.68–1.31)	.731
Hospitalized for sepsis within 14 days	1.89 (1.03–3.47)	.038	3.21 (1.59–6.50)	.001	1.91 (0.98–3.73)	.058
Hospitalized for AKI within 14 days	0.55 (0.23–1.31)	.175	1.53 (0.49–4.79)	.457	0.87 (0.40–1.90)	.727
Death within 28 days	1.44 (1.12–1.85)	.004	1.18 (0.83–1.68)	.353	1.39 (0.93–2.07)	.108

Abbreviations: AKI, acute kidney injury; CI, confidence interval; OR, odds ratio; UTI, urinary tract infection.

### Risk of Adverse Outcomes

Compared with nitrofurantoin, patients prescribed cefalexin, ciprofloxacin, or co-amoxiclav had lower odds of reconsultation and represcription (OR for cephalexin = 0.85, 95% CI = 0.75–0.98, *P* = .020; OR for ciprofloxacin = 0.48, 95% CI = 0.38–0.61, *P* ≤ .001; OR for co-amoxiclav = 0.77, 95% CI = 0.64–0.93, *P* = .006) (Table 2). We found no significant difference in the odds of hospitalization for UTI between patients prescribed nitrofurantoin versus cefalexin, ciprofloxacin, or co-amoxiclav. However, compared with nitrofurantoin, patients prescribed ciprofloxacin had greater odds of hospitalization for sepsis (OR = 3.21, 95% CI = 1.59–6.50, *P* = .001), as did patients prescribed cefalexin (OR = 1.89, 95% CI = 1.03–3.47, *P* = .038). We found no significant difference in the odds of hospitalization for AKI between patients prescribed nitrofurantoin versus cefalexin, ciprofloxacin, or co-amoxiclav. Compared with nitrofurantoin, patients prescribed cefalexin had greater odds of death within 28 days of the UTI (OR = 1.44, 95% CI = 1.12–1.85, *P* = .004).

### Sensitivity Analyses

The association between patients prescribed ciprofloxacin or cefalexin and lower odds of reconsultation and represcription could be due to the significantly increased rates of sepsis hospitalization (ciprofloxacin) and death (cefalexin) in these groups, preventing patients’ re-presenting to primary care. Therefore, we combined these 3 outcomes and found that 7.1% of patients prescribed nitrofurantoin reconsulted or were hospitalized for sepsis or died, compared with 6.3% of patients prescribed ciprofloxacin or cefalexin, with an adjusted OR for the combined outcome of 0.87 (95% CI, 0.78–0.95).

## DISCUSSION

Our results show that patients prescribed cefalexin, ciprofloxacin, or co-amoxiclav had lower odds of reconsultation and represcription. Patients prescribed cefalexin or ciprofloxacin had greater odds of sepsis hospitalization, and those prescribed cefalexin had greater odds of death. Overall, compared with nitrofurantoin, we found no evidence that cefalexin, ciprofloxacin, or co-amoxiclav were associated with a reduction in the risk of UTI-related hospitalization or death.

### Results in Context

The lower odds of reconsultation and represcription among patients prescribed cefalexin, ciprofloxacin, or co-amoxiclav may reflect lower odds of treatment failure. This was in contrast to previous trials that generally showed similar clinical cure rates between narrow and broad-spectrum agents [16, 28, 29]. This association remained significant when we combined the reconsultation and represcription outcome with hospitalization for sepsis or death, suggesting that, despite the higher rates of sepsis/death in the cefalexin/ciprofloxacin group, there remain a group of patients who were less likely to experience treatment failure with these agents. However, we were unable to distinguish whether patients in the nitrofurantoin group who reconsulted and received another antibiotic prescription did so because of an adverse event or intolerance, rather than for treatment failure.

We found increased odds of sepsis in patients prescribed cefalexin or ciprofloxacin. This finding may be due to residual unmeasured confounding because these patients were sicker or had more complicated infection. It may also relate to higher levels of prior fluoroquinolone exposure, previously shown to be associated with increased sepsis risk, possibly due to disruption of the gut microbiome and subsequent dysregulation of the immune response to infection [30].

Our finding of an increased risk of death in patients prescribed cefalexin is intriguing. There are several possible explanations. The antibiotic itself may increase the risk of death, particularly in this cohort, many of whom had multiple comorbidities and were prescribed multiple other drugs. This is not implausible; cefalexin use is associated with antibiotic-associated diarrhea and *Clostridium difficile* infection, which may result in serious and protracted illness in elderly comorbid patients [31]. It may also be due to antimicrobial resistance. For example, the 2017 English Surveillance Programme for Antimicrobial Utilisation and Resistance report showed that 10% of community-acquired *Escherichia coli* UTIs were resistant to cefalexin but only 2% to nitrofurantoin [32]. Finally, some of these findings could again be due to residual confounding. Patients prescribed cefalexin may have been less healthy, presented with more severe illness, and were therefore more likely to experience an adverse outcome irrespective of the prescribed antibiotic. Thus, it may be more appropriate to regard the exposure as a combination of patient and prescription factors, which is why we have related associations to the “patients prescribed cefalexin” rather than the prescription alone.

### Strengths and Weaknesses of This Study

We used data from a general practice database that is broadly representative of the UK population [20]. Cohort entry was dependent on presentation and empirical treatment of UTI in primary care and thus reduced indication bias. We also reduced indication bias by propensity-score matching and achieving adequate balance of baseline characteristics across the groups.

Our study has some limitations. We attempted to capture patients presenting with UTI but had no microbiological data to support this. However, although a limitation, this is also more representative of clinical practice. Our outcomes, particularly sepsis and AKI, relied on coding and were not microbiologically or biochemically confirmed. We were unable to determine precise reasons for reconsultation and represcription and acknowledge that not all of these events may have been due to treatment failure. We were unable to determine antibiotic treatment duration and therefore could not include this potentially important variable in the propensity score model. Based on current definitions [8], some patients may have presented with “complicated” UTI, for which the recommended treatment includes some of the alternative antibiotics assessed. Therefore, we have not commented on the appropriateness (or not) of the prescribed agent. Our findings are based on prescriptions and not on dispensed or ingested drugs. Finally, despite our design, differential coding, indication bias, and residual confounding may have affected our findings.

## CONCLUSIONS

Our findings highlight the challenges associated with selecting antibiotics for older patients with suspected UTI. Compared with nitrofurantoin, we found no evidence that cefalexin, ciprofloxacin, or co-amoxiclav prescribing was associated with a reduced risk of hospitalization or death, suggesting that the perceived aim expressed by clinicians in previous qualitative work was not being achieved, and thus supporting further reductions in prescribing of these agents, even in frailer, sicker patients, especially given their impact on antimicrobial resistance. Future research should explore reasons for continued use of these antibiotics for UTI in primary care and provide clinicians with information on which patients are most likely to benefit from their use.

## Supplementary Data

Supplementary materials are available at *Open Forum Infectious Diseases* online. Consisting of data provided by the authors to benefit the reader, the posted materials are not copyedited and are the sole responsibility of the authors, so questions or comments should be addressed to the corresponding author.

ofz039_suppl_supplementary_material
