# Supplementary material for: Choice of Empirical Antibiotic Therapy and Adverse Outcomes in Older Adults With Suspected Urinary Tract Infection: Cohort Study
Source: Open Forum Infect Dis. 2019 Jan 18;6(3):ofz039. doi: 10.1093/ofid/ofz039 (PMC6411277; doi:10.1093/ofid/ofz039)
Supplement: ofz039_suppl_supplementary_material [file ofz039_suppl_supplementary_material.docx]

Supplementary Appendix 1. Code lists

1.Read code list to identify UTI events in primary care records

| **medcode** | **readterm** |
| --- | --- |
| 10515 | Recurrent urinary tract infection |
| 1572 | Recurrent urinary tract infection |
| 9378 | Recurrent urinary tract infections |
| 150 | Urinary tract infection, site not specified NOS |
| 97002 | Urinary tract infection |
| 107568 | Catheter-associated urinary tract infection |
| 2650 | Chronic urinary tract infection |
| 107843 | CAUTI - catheter-associated urinary tract infection |
| 1289 | Urinary tract infection, site not specified |
| 389 | Cystitis |
| 15074 | Acute cystitis |
| 1353 | Recurrent cystitis |
| 12484 | Cystitis NOS |
| 10857 | Other specified cystitis |
| 34630 | Other cystitis NOS |
| 7579 | Suspected UTI |
| 3306 | H/O: recurrent cystitis |
| 2985 | Recurrent UTI |

| 532 | Dysuria |  |
| --- | --- | --- |
| 667 | Urinary symptoms |  |
| 507 | Haematuria |  |
| 6247 | H/O: haematuria |  |
| 4160 | Frequency of micturition |  |
| 7300 | Suprapubic pain |  |
| 22278 | Frequency of micturition |  |
| 99784 | Lower urinary tract symptoms |  |
| 6161 | Incontinence of urine |  |
| 729 | Urinary frequency |  |

2.ICD-10 codes used to identify hospitalisation-related outcomes

2.1. Hospitalisation for UTI

**Code Description**

N30 Cystitis

N30.0 Acute cystitis

N30.8 Other cystitis

N30.9 Cystitis unspecified

N39.0 Urinary tract infection, site not specified

2.2. Hospitalisation for sepsis

**Code Description**

A41.5 Gram-negative sepsis NOS

A41.8 Other specified sepsis

A41.9 Sepsis, unspecified

A49.9 Bacteraemia NOS

R57.2 septic shock

2.3. Hospitalisation for acute kidney injury

**Code Description**

N17 Acute renal failure

N17.0 Acute renal failure with tubular necrosis

N17.1 Acute renal failure with acute cortical necrosis

N17.2 Acute renal failure with medullary necrosis

N17.8 Other acute renal failure

N17.9 Acute renal failure, unspecified

N19 Unspecified kidney failure
